# The Effect of Attentional Direction on Sub-Stages of Preparing for Motor Skill Execution Across Practice

**DOI:** 10.1177/00315125211009026

**Published:** 2021-04-30

**Authors:** Mengkai Luan, Arash Mirifar

**Affiliations:** 1 Department of Sport and Health Sciences, Technische Universität München, Germany

**Keywords:** motor skill, dual task, attentional direction, preparation phase

## Abstract

While several empirical studies using dual-task methodology have examined the effect of attentional direction on motor skill execution; few have studied the effect of attentional direction on just the preparation phase of motor practice. In this study, via a keying sequence paradigm, we explored processing stages of preparation for a motor skill and disentangled the effect of attentional direction on various stages across practice. First, participants learned two keying sequences (three versus six keys). Then, they practiced the keying sequences in response to corresponding sequence labels under two block-wise alternating dual-task conditions. To dissect the preparation phase into sequence selection and sequence initiation stages, participants received varying amounts of preparation time (0, 300, 900 ms) before a starting signal instructed them to begin sequence execution. In each trial, a tone was paired with one of the three or six keypresses, and participants indicated either the keypress with which the tone was presented (skill-focused dual task) or the tone’s pitch (extraneous dual task) after the sequence execution. We found that attentional direction affected only the sequence selection stage, not the sequence initiation stage. During early practice, compared to drawing attention away from execution, directing attention toward execution led to faster sequence selection. This advantage decreased with practice and vanished during late blocks of trials. Moreover, for the execution phase, relative to directing attention toward execution, drawing attention away from execution led to better performance of keying sequence execution across practice. Thus, attentional direction alone does not fully explain the difference between performance patterns at different skill levels in the dual-task literature; rather, types of motor skills and dual task difficulty levels may also drive performance differences.

## Introduction

According to several theories of motor skill learning (e.g., Anderson, 1982; Fitts & Posner, 1967), attentional requirements for motor skills change with practice and experience. In early learning phases, motor skill execution is supported by a set of unintegrated components of the motor skill that should be kept in working memory and consciously attended to step-by-step (Beilock et al., 2004; Gray, 2004). Gradually, with extended practice, motor skill components are mediated more and more by procedural memory, and the motor skill, therefore, can be executed more automatically with minimum attentional demands (Anderson, 1982; Fitts & Posner, 1967). Such a decrease in attentional demands for executing a motor skill, which is caused by practice, has led researchers to investigate how attentional direction affects motor skill performance across skill levels — either by shifting attention away from execution or by drawing attention toward it. Studies to date, however, have mostly examined attentional direction effects on performance outcomes and, to some extent, on the execution phase (e.g., Beilock et al., 2002, 2004; Gray, 2004; Jackson et al., 2006), while few studies have examined its effect on the preparation phase.

To study the role of attentional direction in motor skill performance, previous studies have used “dual-task methodology” (e.g., Beilock et al., 2002, 2004; Beilock & Gray, 2012; Gray, 2004; Jackson et al., 2006), which refers to experiments in which participants perform a primary task related to the motor skill and, simultaneously, a secondary unrelated task, generally cognitive in nature. Two categories of secondary tasks have been distinguished: (a) a skill-focused dual task that directs attention toward motor skill execution (e.g., reporting the side of the foot contacting the ball while soccer dribbling), and (b) an extraneous dual task that draws attention away from motor skill execution (e.g., identifying irrelevant auditory stimuli). Previous studies using dual-task methodology have revealed that the effect of attentional direction differs across levels of motor skills (e.g., Beilock et al., 2002; 2004; Beilock & Gray, 2012; Castaneda & Gray, 2007; Gray, 2004; Jackson et al., 2006). An extraneous dual task hardly impacted high-skilled performance, but it greatly interfered with low-skilled performance (Beilock et al., 2002, 2004; Jackson et al., 2006). Skill-focused dual tasks, however, impaired high-skilled participants’ performance to a greater degree than that of low-skilled participants (Beilock et al., 2002; Gray, 2004). In other words, low-skilled participants benefit more than high-skilled participants from directing attention toward, rather than away, from motor skill execution; whereas high-skilled participants show the opposite pattern.

For example, Gray (2004) asked participants to complete a motor task of simulated baseball batting under dual-task conditions. During each trial, one of two different tones (250 or 500 Hz) occurred during the swing phase of baseball batting. In the skill-focused dual-task condition, participants were required to verbally indicate whether the tone occurred during the downward or the upward phase of the swing at the instant the tone was presented; in the extraneous dual task, participants were required to indicate whether the tone was the higher or the lower pitched tone at the instant the tone was presented. Novices’ batting performance was better in the skill-focused dual-task condition than in the extraneous dual-task condition, but experts displayed superior batting performance in the extraneous dual-task condition relative to the skill-focused dual-task condition. Similar results have been reported for a golf putting task (Beilock et al., 2002; Beilock & Gray, 2012) and a soccer dribbling task (Beilock et al., 2002; Jackson et al., 2006).

Low-skilled participants are expected to attend to motor skill execution without specific direction to do so, since conscious processing is used to control execution in a step-by-step fashion. Hence, directing attention toward motor skill execution should not be disruptive to their performance. In contrast, when engaging in an extraneous dual task in addition to their primary task, low-skilled participants’ performance is impaired due to insufficient availability of attentional resources. However, for high-skilled participants, the motor skill can be executed automatically without attentional monitoring. Drawing attention away from motor skill execution should not impair their performance, because they can direct most of their attentional resources to process the extraneous dual task. In contrast, directing attention toward motor skill execution would hinder their performance by bringing elements of motor skill back into working memory, resulting in a breakdown of automatic mechanisms (Castaneda & Gray, 2007; Ford et al., 2005).

Most previous studies using dual-task methodology have examined these attentional direction effects on motor skill execution and performance outcomes, leaving still unclear how attentional direction affects the preparation phase of motor skill execution. In the motor skill preparation phase, the individual must select appropriate motor schemas according to proper internal and external cues and then must organize these schemas into a suitable sequence (Jeannerod, 1997). Both motor skill preparation and execution phases are critically important for any motor task that requires rapid responses to signals and a coordination of multiple effectors (Ille et al., 2013). However, most primary motor tasks in dual-task research have been continuous or self-paced tasks that participants began executing when they were ready and that did not require a short reaction time.

A recent study (Luan et al., 2020) proposed an advantage for drawing attention toward motor skill execution for the preparation phase in early practice but suggested that this advantage diminished with practice. Luan et al. (2020) had participants learn two keying sequences (a three-key and a six-key sequence, labeled X and Y respectively) and then practice them in response to the label cue (X or Y) under two different dual-task conditions. During each keying sequence execution, a tone was presented along with one of the keypresses. In the extraneous dual-task condition, participants finished the keying sequence execution and then indicated whether the tone was of a higher or lower pitch. In the skill-focused dual-task condition, they completed the keying sequence and then indicated the keypress with the tone presentation. Reaction times (defined as the time between the presentation of the sequence label cue and the first keypress) were faster in the skill-focused dual-task condition than in the extraneous dual-task condition, and the gap between reaction times in the two dual-task conditions decreased and diminished with practice.

Although Luan et al. (2020) results clearly showed how attentional direction influences the preparation phase of a keying sequence across practice, these results did not differentiate attention direction effects on sub-stages of the preparation phase. In a traditional information-processing stage theory of keying sequences, both sequence selection and sequence initiation can be treated as separate serial sub-stages of a cognitive process encapsulated within the preparation phase (Dudman & Krakauer, 2016; Kunde et al., 2004; Spijkers & Walter, 1985; Stöcker & Hoffmann, 2004). Sequence selection precedes sequence initiation and provides the signal for executing the keying sequence, corresponding with the stimulus. Sequence initiation initiates the selected keying sequence and occurs before the execution phase. Luan et al. (2020) included both selection and initiation stages within the reaction time measure, meaning that this study could not distinguish which sub-stage was influenced by attentional direction.

Therefore, in the present study, our main purpose was to better understand how attentional direction influences the preparation phase of motor skill execution across practice. We intended to disentangle attentional direction’s influence on selection and initiation stages of keying sequence. This study’s experimental design was nearly identical to Luan et al. (2020) paradigm, with one exception. We used different stimulus onset asynchronies (SOAs; 0, 300, 900 ms) between the presentation of the sequence label cue and the starting signal in order to provide participants with varying amounts of preparation time. We used the participants’ reaction time (RT), defined as the time between the starting signal and the first keypress and differing from that in Luan et al. (2020), to indicate performance of the separate sub-stages of the preparation phase of the keying sequence. We compared the difference in RT between dual-task conditions in different SOA conditions. The rationale for manipulating SOA was that if increasing SOA between the presentation of the sequence label cue and the starting signal has no differential influence on RT differences between dual-task conditions, then attentional direction differentially affects the sequence initiation stage, not the sequence selection stage. If increasing SOA decreases RT differences between dual-task conditions, then attentional direction affects sequence selection and/or sequence initiation stages. If sufficiently long SOAs result in no difference in RTs between dual-task conditions, then attentional direction affects only the sequence selection stage, not the sequence initiation stage. We also used movement duration (MD), also known as “movement time,” (the same as in Luan et al., 2020) to indicate the performance of the execution phase of the keying sequence, since we also wanted to explore whether different SOA conditions affected performance in the execution stage.

## Method

### Participants

Thirty-two undergraduate and graduate students (15 male, 17 female) with an age range between 19 and 28 years (*M* = 23.2, *SD* = 2.1) participated in this study for extra credit. All participants were right-handed and naive to dual-task methodology and to the keying sequence task. Invited to the lab individually, they first received a short tour. They were then informed about the experimental procedure, their rights, and the anonymity of experimental data. Finally, they were asked to sign an informed consent form, according to Declaration of Helsinki guidelines. For reasons explained below, one participant’s data was excluded from analysis. The study did not involve any invasive or potentially dangerous methods. According to the German Science Foundation and the guidelines of the first author’s institution, formal ethical approval was not required.

### Apparatus

Presentation of stimuli and registration of responses were achieved by MATLAB 2017 b (the MathWorks Inc., Natick, MA, USA) and the Psychtoolbox-3 extensions (http://psychtoolbox.org/) on a PC (FUJITSU DTF, Intel (R) Core (TM) i7-7700, 3.60 GHz CPU, 16 GB RAM, 64-bit Windows 10). Stimuli were presented on a 17-inch Dell E1715S monitor, approximately 50 cm in front of participants. The screen’s spatial resolution was set to 1024 × 768. In the experiment, the screen’s background was black, and the instructions were in white 16-point Arial font. Tones were presented via two loudspeakers located approximately 20 cm bilaterally from the monitor.

The experiment employed one custom-built keyboard, consisting of two “BLACK BOX TOOLKIT” four-button response pads. Eight keys of the two response pads were relocated as a horizontal line across the keyboard’s vertical center and labeled with eight letters (A, S, D, F, G, H, J, and K). Throughout the experiment, participants rested their left index, middle, ring, and little fingers on buttons corresponding to F, D, S, and A keys, respectively, and rested their right index, middle, ring, and little fingers on buttons corresponding to G, H, J, and K keys, respectively.

### Study Design and Procedure

**Figure 1.**
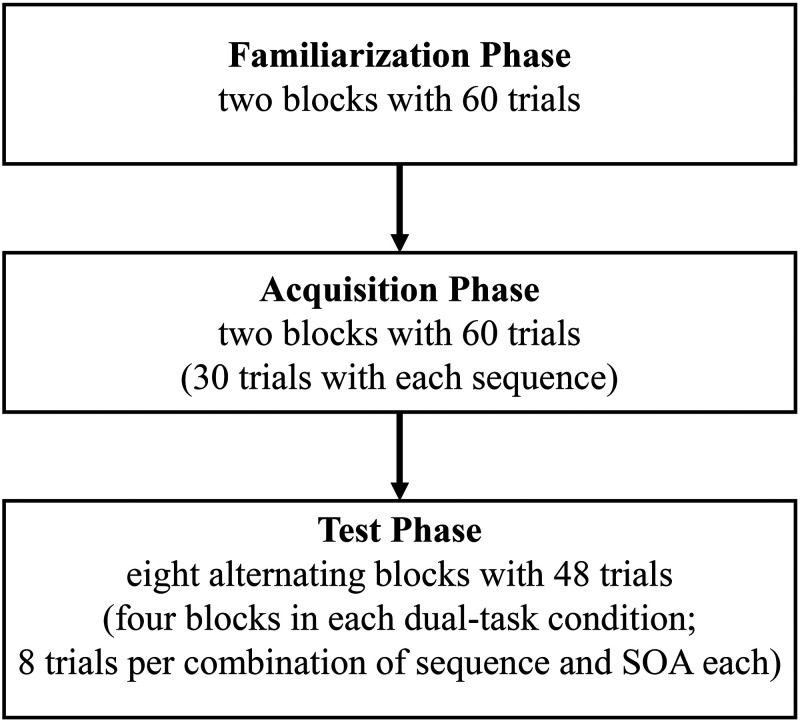
Flow Chart of the Procedure.

**Figure 2.**
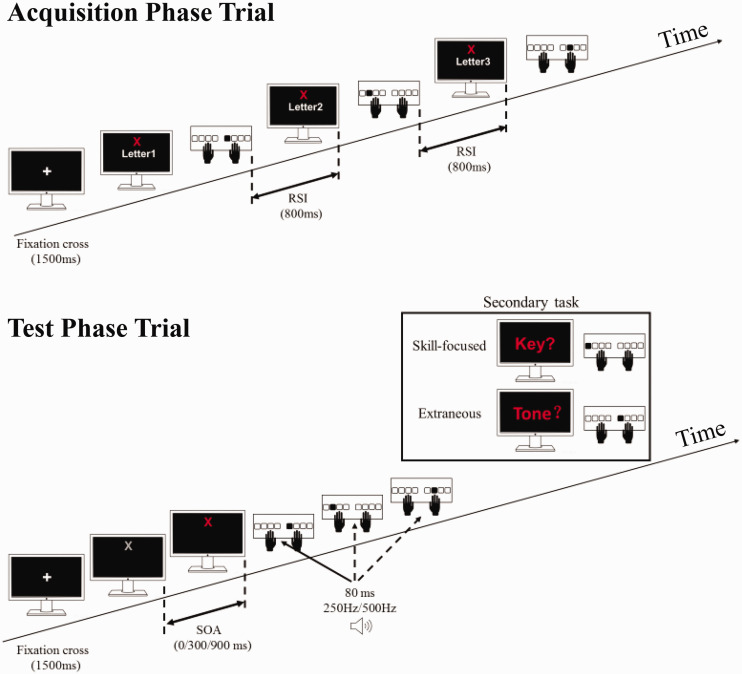
Schematic Illustration of the Display and the Timing of Events in the Acquisition Phase and the Test Phase. Note: We used the three-key sequence labeled X trials as examples. They could also be the six-key sequence labeled Y trials.

The primary task in the present study was to learn two bimanual keying sequences and practice them in two dual-task conditions (skill-focused and extraneous) — a modified design from that of Luan and colleagues (2020). The experiment consisted of three phases (the familiarization phase, the acquisition phase, and the test phase). At the beginning of each phase, we presented instructions as text on the screen. Figure 1 presented the flow chart of the procedure. Figure 2 displayed the trial types in the acquisition phase and the test phase.

### Familiarization Phase

Participants learned about the apparatus and stimuli during the familiarization phase, which consisted of two blocks with 60 trials each. In each trial, participants were presented one of the six letters (S, D, F, G, H, or J) in white 16-point Arial font at the monitor’s center, and they were to press the assigned key as quickly as possible. Letters were presented randomly, each occurring equally often. When a participant pressed the correct key, after an 800-ms interval, the next trial presented the next letter stimulus. When a participant pressed a wrong key, the message ‘‘Error’’ appeared for 700 ms at the bottom of the screen. Then, after an 800-ms interval, the next trial began.

### Acquisition Phase: Learning Keying Sequences

In the second phase, participants learned two keying sequences in response to single sequence-specific letters, a short one labeled “X” and a long one labeled “Y.” With the letters “R,” “M,” and “I” standing for ring, middle, and index fingers and lowercase standing for the left hand, for all participants, the short sequence was I-r-M (learned as G-S-H) and the long sequence was r-I-i-M-m-R (learned as S-G-F-H-D-J).

In this phase, the term “trial” indicated an entire sequence. In each trial, after presentation of a white fixation cross (approximately 0.70° of visual angle) for 1,500 ms, a sequence label (“X” or “Y”) in white 60-point Arial font appeared throughout the entire trial in the monitor’s upper half. Then the first letter of the sequence with that label was presented at the center point, and participants pressed the corresponding key, as in the familiarization phase. When the participant pressed the correct key, the next letter of that sequence appeared after a response-to-stimulus interval (RSI; the delay between a participant’s response and the next stimulus’s appearance) of 800 ms. RSI manipulation prevented participants from practicing fast execution of keying sequences in this phase but enabled them to build up central-symbolic representations of the two sequences (Luan et al., 2020). If a wrong key was pressed, the message ‘‘Error’’ appeared for 700 ms at the bottom of the monitor before the RSI. After the entire sequence ended, the sequence label cue disappeared, and the next trial began. Participants were instructed to focus on memorizing the two sequences and minimizing mistakes, not necessarily to react fast because they would later be asked to execute keying sequences based on the stimulus of the sequence label alone without a key-specific cue. This phase contained two blocks each consisting of 60 trials (30 trials with each sequence). Sequence order was randomized across each block. After each block, participants’ error rate for that block appeared on the monitor for five seconds.

### Test Phase: Performing in Dual-Task Conditions

The test phase consisted of eight alternating blocks, each with one of the dual-task conditions (skill-focused or extraneous); the starting condition was counterbalanced across participants. Each block began with an instruction on the screen about the block’s dual-task version. In each test phase trial, a fixation cross appeared at the monitor’s center for 1500 ms, after which the sequence-specific cue (“X” or “Y”) appeared. The three different SOA conditions were 0 ms, 300 ms, and 900 ms. In trials with an SOA different from 0 ms, the sequence cue was first presented in white. After the SOA had passed, the color of the sequence cue changed to red. In trials with 0 ms SOA, the sequence cue letter was presented in red directly. Participants were supposed to initiate immediately and execute the corresponding keying sequence as quickly as possible without mistakes after the SOA passed (i.e., the cue color changed to red). Note that in this phase, key-specific cues did not appear. Either one 80-ms low-pitched tone (250 Hz) or one 80-ms high-pitched tone (500 Hz) was presented to participants with the pressing of one of the three or six keys in each trial. The keystroke with which the tone was paired and the tone’s frequency were randomly, but equally, distributed. At the beginning of the test phase, for familiarization, the two tones were presented five times in alternation.

In the skill-focused dual-task condition, participants monitored which keypress was accompanied by the tone. After completing each sequence, they reported the keystroke by pressing the corresponding key (S, D, F, G, H, or J). In the extraneous dual-task condition, participants carefully monitored the tone’s frequency. After completing each sequence, they identified whether the tone was low-pitched or high-pitched by pressing a corresponding key (“A” for low frequency and “K” for high frequency). After participants answered the question about the concurrent cognitive task, the next trial started automatically. When a wrong key was pressed during the keying sequence execution, the word “Error” appeared (red 16-point Arial) for 700 ms at the bottom of the screen. Then, the current trial was aborted, and the next trial began.

Each block consisted of 48 trials (24 with each sequence). The combination of sequence and SOA varied randomly, but occurred equally, often in each block; that is, there were eight trials per combination of sequence and SOA. At the end of each block, the error rate and the mean RT of the keying sequence task for that block appeared onscreen for five seconds. Each block was followed by a 30-second break except for block 4 (60 seconds).

### Data Processing and Statistical Analysis

Only test phase trials were analyzed. One male participant was not included in the analysis due to failure to remember the two sequences and therefore an inability to complete the test phase. Each participant’s RT and MD were recorded for each trial. RT was measured as the time from the moment the sequence cue turned red to the first keypress; MD was measured as the time from the first to the last keypress. To reduce the influence of transitioning from one dual-task condition to another (i.e., sequentially), each block’s first two trials were excluded from analysis. Furthermore, trials containing a wrong keypress were considered erroneous and excluded. In the end, for each participant, more than 95% of “X” sequence trials and 90% of “Y” sequence trials remained. We analyzed mean RT and MD per participant, sequence, dual-task condition, SOA, and block by using a 2 (Sequence: 3-key vs. 6-key) × 2 (Dual-task: skill-focused vs. extraneous) × 3 (SOA: 0 ms, 300 ms, 900 ms) × 4 (Block) analyses of variance (ANOVAs) with repeated measurement. Where significant Dual-task × SOA × Block interaction occurred, separate ANOVAs with Sequence, Dual-task, and Block as factors for each SOA condition were conducted to examine Dual-task × Block interaction in different SOA conditions. We applied the Greenhouse–Geisser correction when Mauchly’s test indicated that the assumption of sphericity was violated. Estimated marginal means and differences between estimated marginal means were calculated to represent main effects and interaction effects.

## Results

### Reaction Time

**Figure 3.**
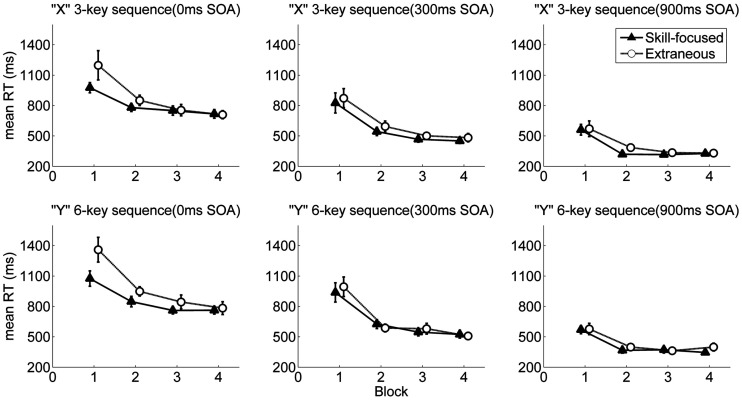
Reaction Time as a Function of Block, Dual Task, SOA, and Sequence. Note: Error bars represent standard errors.

The ANOVA revealed a main effect of Block, *F*(1.16, 34.86) = 43.72, *p* < 0.001, ${\text{η}}_{p}^{2}$ = 0.59, indicating that RTs decreased with practice. There was a main effect of Sequence, *F*(1, 30) = 12.75, *p* = 0.001, ${\text{η}}_{p}^{2}$ = 0.30, showing that RTs in the three-key sequence were shorter than RTs in the six-key sequence. There was also a main effect of SOA, *F*(1.50, 45.11) = 253.85, *p* < 0.001, ${\text{η}}_{p}^{2}$ = 0.89, such that RTs decreased with increasing SOA (0 ms SOA: *M* = 883 ms; 300 ms SOA: *M* = 628 ms; 900 ms SOA: *M* = 409 ms). A significant SOA × Block interaction, *F*(2.80, 83.94) = 6.21, *p* = 0.001, ${\text{η}}_{p}^{2}$ = 0.17, indicated that SOA effect on RT decreased with practice. Statistical significance was observed in the main effect of Dual-task, showing faster RT in the skill-focused dual-task condition (*M* = 615 ms) than in the extraneous dual-task condition (*M* = 664 ms), *F*(1, 30) = 4.22, *p* = 0.049, ${\text{η}}_{p}^{2}$ = 0.12. The main effect of Dual-task was qualified by a significant Dual-task × SOA interaction, *F*(1.79, 53.76) = 7.41, *p* = 0.002, ${\text{η}}_{p}^{2}$ = 0.20, showing that RT difference between the two dual-task conditions decreased with increased SOA (differences between estimated marginal means for dual-task conditions across SOA conditions were 97, 35, and 23 ms for 0-ms, 300-ms, and 900-ms SOA conditions, respectively). In addition, a significant Dual-task × SOA × Block interaction, *F*(2.65, 79.56) = 3.2, *p* = 0.033, ${\text{η}}_{p}^{2}$ = 0.10, suggested that RT difference between the two dual-task conditions in early practice blocks decreased more with increased SOA. All other interactions were nonsignificant, *p*s > 0.19 (Figure 3).

To investigate this three-way interaction further, we conducted three separate ANOVAs for the three SOA conditions with Sequence, Dual-task, and Block as factors. In the 0-ms SOA condition, a significant main effect of Dual-task was observed, *F*(1, 30) = 11.75, *p* = 0.002, ${\text{η}}_{p}^{2}$ = 0.28, showing faster RT in the skill-focused dual-task condition (*M* = 834 ms) than in the extraneous dual-task condition (*M* = 931 ms). The Dual-task × Block interaction was also significant, *F*(1.50, 45.05) = 3.74, *p* = 0.043, ${\text{η}}_{p}^{2}$ = 0.11, such that RT difference between the two dual-task conditions decreased with practice (differences between estimated marginal means for dual-task conditions across practice were 253, 76, 44, and 7 ms for Blocks 1–4, respectively). Further planned comparisons detected that RT in the skill-focused dual-task condition was faster than the extraneous dual-task condition in the first two blocks, *F*s(1, 30) < 6.49, *ps* > 0.016, ${\text{η}}_{p}^{2}$s > 0.18, whereas no RT differences were found for Blocks 3 and 4, *F*s(1, 30) < 1.09, *p*s > 0.30. However, in the other two SOA conditions (300 ms and 900 ms), ANOVAs showed that neither the main effect dual-task nor the dual-task × block interaction approached significance, *p*s > 0.24.

### Movement Duration

**Figure 4.**
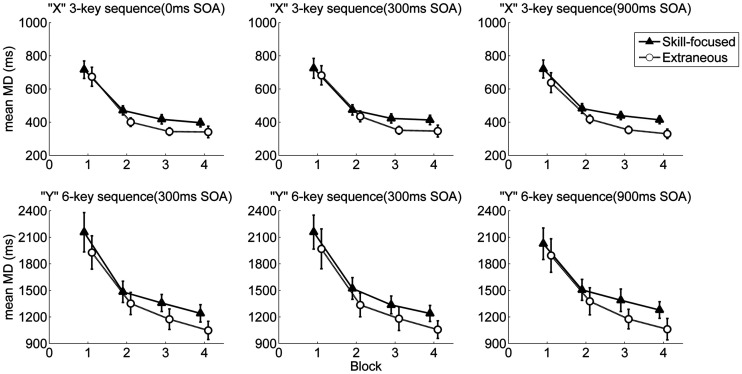
Movement Duration as a Function of Block, Dual Task, SOA, and Sequence. Note: Error bars represent standard errors.

The ANOVA showed a significant main effect of Sequence, *F*(1, 30) = 90.86, *p* < 0.001,$\;{\text{η}}_{p}^{2}$ = 0.75, and a significant main effect of Block, *F*(1.08, 32.46) = 65.34, *p* < 0.001,$\;{\text{η}}_{p}^{2}$ = 0.69, indicating that MD decreased with practice and that it was shorter in the three-key than in the six-key sequence. A significant Sequence × Block interaction, *F*(1.15, 34.38) = 30.97, *p* < 0.001, ${\text{η}}_{p}^{2}$ = 0.51, indicated that the difference in MD between the two sequences decreased with practice (differences between estimated marginal means for sequences across practice were 1530, 982, 880, and 781 ms for Block 1–4, respectively). There was also a significant main effect of Dual-task, *F*(1, 30) = 7.08, *p* < 0.012,$\;{\text{η}}_{p}^{2}$ = 0.19, indicating that MD was generally slower in the skill-focused dual-task condition (*M* = 1032 ms) than in the extraneous dual-task condition (*M* = 911 ms). Moreover, a significant Dual-task × Sequence interaction, *F*(1, 30) = 4.19, *p* = 0.049, ${\text{η}}_{p}^{2}$ = 0.12, suggested that the MD difference between the two dual-task conditions was larger in the six-key than in the three-key sequence. The main effect of SOA was not statistically significant, and neither were other interactions, *p*s > 0.12 (Figure 4).

## Discussion

The present study examined the effect of attentional direction on sub-stages of the preparation phase of a motor skill — consisting of selection and initiation — for keying a sequence under a dual-task paradigm across practice. Our results indicated that in the 0-ms SOA condition, RTs in the skill-focused dual-task condition were initially faster than RTs in the extraneous dual-task condition, and the gap between RTs under the two different conditions gradually decreased with practice and vanished in the final block. This part of our results replicated previous findings by Luan et al. (2020). Most importantly, there was no difference in RT between the two dual-task conditions in the 300-ms and 900-ms SOA conditions, that is, the RT advantage in the skill-focused condition was eliminated by increasing preparation time. In addition, we analyzed the effect of attentional direction on the execution phase of keying sequences in different SOA conditions. We found that for MD, SOA conditions had no differential influence on the effect of attentional direction. Furthermore, results showed that directing attention toward execution generally impaired execution more than shifting attention away from execution, thus also replicating MD results in Luan et al. (2020).

Results for the preparation phase of the keying sequence showed that during early practice, the skill-focused dual-task condition was more beneficial than the extraneous dual-task condition when the SOA was set to 0 ms. These results indicated that directing attention toward, rather than away from, execution benefited the preparation phase of keying sequences; however, with increased SOA (from 0 to 300 and 900 ms), there were no differences in RT performance between dual-task conditions across practice. This indicates that the disadvantageous influence of shifting attention away from execution during early practice can be counteracted with additional preparation time. In turn, this strongly suggests that faster RT induced by the skill-focused dual-task condition is due to facilitation of sequence selection, not sequence initiation that occurs after presentation of the starting signal (Kunde et al., 2004; Stöcker & Hoffmann, 2004).

These results suggest that, during early practice, participants selected the keying sequence to respond to a sequence label cue by searching and retrieving explicit knowledge of the keying sequence (this sequence’s series of letters) from memory (Anderson, 1982; Beilock & Gray, 2012; Fitts & Posner, 1967). Search and retrieval processes required working memory engagement (Thompson, 2013). Meanwhile, participants had to maintain the secondary task requirement in working memory (Koch et al., 2018). The skill-focused dual-task required them to pay attention to execution, and the extraneous dual-task required them to shift attention away from execution and pay attention to an extraneous stimulus (Luan et al., 2020). Clearly, novices were inclined to focus their attention on the motor skill itself and dismiss irrelevant information while preparing their motor skill execution (Anderson, 1982; Fitts & Posner, 1967). Therefore, during early practice, maintaining an attentional focus on an extraneous stimulus might require their greater effort and entail higher working memory engagement than maintaining attention to keying sequence execution, because the latter fits their attention’s natural direction (Marchant et al., 2009). Consequently, when the SOA was set to 0 ms, the sequence selection sub-stage of the preparation phase of the keying sequence was slower in the extraneous dual-task condition because more cognitive resources were needed for maintaining the requirement of extraneous dual task and fewer cognitive resources were available for motor skill preparation. Across time, with sufficient practice, explicit knowledge of keying sequences became part of a mental representation of the sequence label cue (Luan et al., 2020; Thompson, 2013). In this circumstance, the keying sequence could be selected without the need for as many cognitive resources. Thus, the sequence selection would not be affected by the dual-task conditions. As a result, differences in RTs caused by dual-task conditions in trials with 0-ms SOA disappeared with practice.

Regarding the execution phase of the keying sequence, the analysis of MD indicated that keying sequence execution in the skill-focused dual-task condition was generally slower compared to the extraneous dual-task condition, independent of practice and preparation time. This result aligns with MD results in Luan et al. (2020). However, this result is inconsistent with typical low-skilled performance in the dual-task literature. Inexperienced performers have typically displayed superior performance in the skill-focused dual-task condition relative to the extraneous dual-task condition (e.g., Beilock et al., 2002; 2004; Gray, 2004). Two, not mutually exclusive, explanations may explain these contradictory results. First, given that the keying sequence is itself a high-speed motor task, deciding with which keypress a tone has been paired during such a fast keying sequence execution is difficult and requires significant attention, even during early practice. At the beginning of practice, participants attend to components of motor skill execution (Masters, 1992, 1993) and use a consciously controlled approach to execute keying sequences (Anderson, 1982; Fitts & Posner, 1967). Although controlled attentional processes take time to execute (Posner & Snyder, 1975), the speed of keying sequence execution is still too fast for assessing the keypress according to tone in the skill-focused dual-task condition. Therefore, to assess the keypress correctly, participants might actively slow keying sequence execution. Additionally, because the skill-focused dual-task requires large amounts of attention, attentional resources should be partly occupied by the skill-focused dual-task, leading to fewer available resources for keying sequence execution and, hence, a relatively slow keying sequence execution in the skill-focused dual-task condition (Luan et al., 2020).

Another explanation might be the extraneous dual task’s simplicity. From a skill acquisition and automaticity standpoint, extraneous dual-task impairment in novice performance results from insufficient available attentional resources to support concurrent motor skill execution and dual-task performance (Beilock et al., 2004). This study’s extraneous dual task presented only two possible tones. Arguably, such a simple design does not demand such substantial attentional resources as to prevent novices from attending to execution and to interfere with their motor skill execution (Gabbett & Abernethy, 2012). Thus, in our study, participants could execute the keying sequence without interruption by the extraneous dual task even at the beginning of practice.

Our MD results revealed the possibility that skill-focused dual task can cause interference with motor skill execution, leading to degraded performance during early practice. Both explanations emphasize that attentional direction manipulated by dual tasks alone does not fully explain the difference between performance patterns at different skill levels seen in the dual-task literature; rather, types of motor skill and difficulty levels of dual tasks could also drive performance differences (Raisbeck & Diekfuss, 2015).

## Limitations and Directions for Future Research

The scope of this study was limited in terms of participants' proficiency, as it is not clear which level of skill they reached after the acquisition phase. Although we set an 800-ms RSI to prevent participants from quickly executing the keying sequence, participants might still have gained some proficiency in this motor skill (Luan et al., 2020). Perhaps, for the execution phase, only novices or beginners without proficiency profit from directing attention toward execution (e.g., Beilock et al., 2004; Gray, 2004). Future investigations should thus find a way to quantify levels of expertise across practice.

Another limitation of this study was the simplicity of the extraneous dual task. It is possible that the extraneous dual task used in this study was not challenging enough to lead a cognitive-motor interference with keying sequence execution (Gabbett & Abernethy, 2012). Future research might consider using sufficiently more difficult extraneous dual tasks to see whether the performance patterns at different skill levels were influenced. Using different extraneous dual tasks with different attentional resources demand would aid in the understanding of the role of difficulty levels of dual tasks in motor skill execution in dual-task conditions.

A further aspect, possibly limiting the extent to which conclusions might be generalized to other conditions or motor skills, was our somewhat reductionist approach. First, we took a serial-processing stand, assuming that the mental process can be divided into strictly independent sequential stages (e.g., Sanders, 1980). Secondly, we investigated a fairly simple ballistic, discrete motor skill. Modern models of decision making, however, indicate not only parallel processing (e.g., action selection and initiation, where different “stages” might interact; Hommel et al., 2001), but also allow for “early” processes to continue and for action selection information processing to be active until the very end of an action (Wispinski et al., 2018). Future research using different paradigms (e.g., reaching or point tasks) is needed in order to explore the effect of attentional direction on such interactions and continued processing.

Investigations of the neural mechanisms underlying sequence learning and execution are also strongly recommended. This type of research would benefit from employing brain imaging techniques, such as functional Magnetic Resonance Imaging (fMRI), magnetoencephalography (MEG), and electroencephalography (EEG) to shed light on brain activity during motor skill learning and performance in dual-task conditions. For example, EEG studies have already shown that the power of neural oscillation in the range of high-alpha in the left temporal region (e.g., Kerick et al., 2004) and the co-activation (coherence) between the left temporal region and the frontal midline region at the high-alpha frequency bandwidth (e.g., Deeny et al., 2003; Zhu et al., 2011) reflect the conscious involvement and attentional demands during motor skill learning and performance (Bellomo et al., 2018; Zhu et al., 2011). Because the dual-task methodology manipulates the direction of attention and influences the conscious involvement in motor skill learning and performance (Beilock & Gray, 2012), these measures of brain activity could also be sensitive to dual-task conditions.

## Conclusion

In sum, the current study showed the effect of varying attentional direction on the different processing in sub-stages of the motor skill preparation phase across practice, using a keying sequence paradigm. Our results suggest that both the amount of practice and the sub-stages of the preparation phase can influence the effect of attentional direction. Moreover, we demonstrated that for the execution phase, attentional direction alone does not fully explain the general performance pattern seen in the dual-task literature; rather, types of motor skills and difficulty levels of dual tasks could also drive performance differences. To dissect different processing stages of a motor skill is a new perspective in dual-task research. This study furthered our knowledge of the effects of dual-task attentional manipulations, and this knowledge will help enhance performance at different stages of information processing. Future research that incorporates not only complex human motor skills with high ecological validity but also information on preparation phase brain activity will provide a more complete picture.
